# Editorial

**Published:** 1847-09

**Authors:** 


### THE MEDICAL EXAMINER.

PHILADELPHIA, SEPTEMBER, 1847.

### NATIONAL MEDICAL CONVENTION.

The Report of the Committee on preliminary education will be found on another page, to which we invite attention. The Committee justly observe that, "entirely destitute of the means of legal compulsion, and depending for success, as the Convention must, solely upon the force of professional and public opinion, nothing could be hoped from a standard above the circumstances of the country and the times," &c.

This is undoubtedly a correct view of the case; at the same time, when about to erect a standard, one a little more elevated might as well perhaps have been indicated, as where an object is distinctly placed before the eye as a *sufficient* qualification, few will transcend, while many will fall short of the mark. Nevertheless, it is greatly to be hoped that private preceptors, on whom "*the chief responsibility* rests," as observed by the Committee, will look to the recommendation. While they encourage young men to enter their offices who are unprepared for the proper prosecution of the study of medicine, with what reason can it be expected that the medical colleges will spurn them? To take a young man's fee and keep him two or three years in his preceptor's office, and then, when he applies to enter college, say to him, "go back to your primer, sir, you are not prepared for the study of medicine," would be rank injustice. It is the duty of every preceptor to inquire into the qualifications of a student, natural and acquired, before receiving him into his office, and honestly inform him of what is requisite. Let this be done, and the friends of reform will find that little more will remain to contend for. One who is possessed of a good education has greater means and higher motives to spur him on in his pursuits, and will be likely to select the best schools and emulate the highest examples.

### THE CONCOURS IN FRANCE.

Among other reforms recently advocated, the French practice of concouring for professorships has of late been highly commended by some of our brother journalists. In fact, we at one time advocated it ourselves, but afterward came to learn that its practical operation was not quite equal to its promises in theory. We understood from different sources, that instead of affording a true test, positive and relative, of the fitness of a candidate, it was liable to great abuses, and often led to deception: it was discovered, indeed, that many things were necessary fully to qualify a man to occupy a professor's chair beside those elicited by the concour. Our brethren of the Buffalo Medical Journal and of the St. Louis Medical and Surgical Journal, with many others, will be surprised to read the following, which we extract from the London Medical Gazette of the 16th of July.

"*Abolition of the Concours in France.*—The Chamber of Peers has come to a vote by which the system of election by Concours in France is abolished. Some of the noisy advocates of this electioneering practice are about to present a protest to the Chamber of Deputies against this vote, and to require a restoration of their favorite panacea for bringing out professional talent! But the feeling of the most eminent and experienced men in the profession is decidedly against the re-establishment of this system."

The introduction of this system into France some years since was hailed as the greatest improvement—as closing the door for ever upon favouriteism, and opening the portals wide to genius and learning! And now it is discovered that common sense and business qualifications are quite as necessary as genius and great learning, and that these are not always combined in the same individual. *Change*, in fact, has not turned out to be *improvement*. Not only the professors of the colleges, but likewise the physicians and surgeons of the hospitals, in France, were chosen by concour, and consequently the strife was going on all the time. "Thus," says the Gazette, "last year there were two vacancies for the situation of surgeons to the hospitals of Paris; *there were thirty-two candidates, and the concours lasted five months!*" Nothing could demonstrate the impracticability of such a system better than this fact.

---

### YELLOW FEVER.

Yellow Fever is prevailing in New Orleans more extensively than for a number of years past. On the 19th of last month, the interments of those who died of this disease amounted to seventy-seven, which, considering that a very large number of the inhabitants are always absent during the summer months, and many more fly on the first announcement of the fever, must be regarded as a very large proportion to the number of inhabitants.

At Vera Cruz, also, the vomito, or yellow fever, continues to prevail, but attended with less mortality, we should infer from what we see in the newspapers, than at New Orleans.

---

SHIP FEVER.

Typhus, or Ship Fever, continues to prevail to some extent among the emigrants to our principal ports, more especially those from Ireland. The greater abundance of food, however, will soon effect a change—in fact, has already materially lessened the number of cases arriving in this country as well as in Europe. Whenever the disease has appeared among others than emigrants in this country, it has been only such as were particularly exposed to it, as the inmates of the houses of the sick, and their attendants, including nurses and physicians. Several valuable lives among the latter have been lost, especially in New York and Canada.

